# Maritime Halophyte Species from Southern Portugal as Sources of Bioactive Molecules

**DOI:** 10.3390/md12042228

**Published:** 2014-04-10

**Authors:** Maria João Rodrigues, Katkam N. Gangadhar, Catarina Vizetto-Duarte, Sileshi G. Wubshet, Nils T. Nyberg, Luísa Barreira, João Varela, Luísa Custódio

**Affiliations:** 1 Centre of Marine Sciences, Faculty of Sciences and Technology, University of Algarve, Ed. 7, Campus of Gambelas, Faro 8005-139, Portugal; E-Mails: mary_p@sapo.pt (M.J.R.); gangadharkatkam@gmail.com (K.N.G.); catarina.vizetto@gmail.com (C.V.-D.); lbarreir@ualg.pt (L.B.); jvarela@ualg.pt (J.V.); 2 Instituto de Tecnologia Química e Biológica, Universidade Nova de Lisboa, Avenida da Republica, Oeiras P-2780-157, Portugal; 3 Department of Drug Design and Pharmacology, Faculty of Health and Medical Sciences, University of Copenhagen, Copenhagen DK-2100, Denmark; E-Mails: sgw@sund.ku.dk (S.G.W.); nn@sund.ku.dk (N.T.N.)

**Keywords:** antioxidant, anti-inflammatory, cytotoxicity, halophytes, juncunol, *Juncus acutus*

## Abstract

Extracts of five halophytes from southern Portugal (*Arthrocnemum macrostachyum*, *Mesembryanthemum edule*, *Juncus acutus*, *Plantago coronopus* and *Halimione portulacoides*), were studied for antioxidant, anti-inflammatory and *in vitro* antitumor properties. The most active extracts towards the 1,1-diphenyl-2-picrylhydrazyl (DPPH) radical were the methanol extracts of *M. edule* (IC_50_ = 0.1 mg/mL) and *J. acutus* (IC_50_ = 0.4 mg/mL), and the ether extracts of *J. acutus* (IC_50_ = 0.2 mg/mL) and *A. macrostachyum* (IC_50_ = 0.3 mg/mL). The highest radical scavenging activity (RSA) against the 2,2′-azino-*bis* (3-ethylbenzothiazoline-6-sulphonic acid) (ABTS) radical was obtained in the ether extract of *J. acutus* (IC_50_ = 0.4 mg/mL) and *H. portulacoides* (IC_50_ = 0.9 mg/mL). The maximum total phenolic content (TPC) was found in the methanol extract of *M. edule* (147 mg gallic acid equivalents (GAE)/g) and in the ether extract of *J. acutus* (94 mg GAE/g). Significant decreases in nitric oxide (NO) production were observed after incubation of macrophages with lipopolysaccharide (LPS) and the chloroform extract of *H. portulacoides* (IC_50_ = 109 µg/mL) and the hexane extract of *P. coronopus* (IC_50_ = 98.0 µg/mL). High *in vitro* cytotoxic activity and selectivity was obtained with the ether extract of *J. acutus*. Juncunol was identified as the active compound and for the first time was shown to display selective *in vitro* cytotoxicity towards various human cancer cells.

## 1. Introduction

Halophytes are highly salt tolerant plants that can be found in sand dunes or rocky coasts, saline depressions or inland deserts, and in marine environments such as coastal salt marshes [1,2]. In order to withstand the often unfavorable conditions of these locations (e.g., high salinity and UV-irradiation levels), halophytic species have developed several physiological traits that allow them to retain and acquire water, protect cells from the damage caused by the accumulation of reactive oxygen species (ROS), and maintain ion homeostasis [2,3,4,5,6]. These traits include the biosynthesis of different primary and secondary metabolites, such as vitamins, terpenoids, phenolics, polysaccharides and glycosides, which display several biological activities, including antioxidant, antimicrobial, anti-inflammatory, and antitumoral, and thus they can be crucial for the prevention of a variety of diseases as, for instance, cancer, chronic inflammation and cardiovascular disorders.

Several halophytic plants have been used in traditional medicine. Representative examples are the treatment of microbial infections (e.g., *M. edule*, Aizoaceae), to reduce blood pressure (*Salsola kali* L., Chenopodiaceae) or in cancer treatment (*Artemisia scopariae* Waldst. and Kit., Asteraceae), and in many cases *in vitro* studies have confirmed these ethnopharmacological uses [2]. Having in mind the high diversity of halophytes (*i.e.*, more than 2500 species have been identified so far), and the relatively small number of bioactive compounds identified, it is easy to understand why halophytes are considered to be an almost unexploited reservoir of novel bioactive molecules, or as novel sources for known compounds. In this context, and following our interest in bioactive compounds present in marine organisms and in plants living in habitats with a strong marine influence, the *in vitro* antioxidant, anti-inflammatory and antitumoral activities of five maritime halophyte species common in the southern parts of Portugal—*A. macrostachyum*, *M. edule*, *J. acutus*, *P. coronopus* and *H. portulacoides*—were evaluated*.* The bioactive compound present in the most active species (*J. acutus)* was isolated and identified and its cytotoxicity and selectivity is reported here for the first time.

## 2. Results and Discussion

### 2.1. RSA against DPPH (1,1-Diphenyl-2-picrylhydrazyl) and ABTS 2,2′-Azino-bis (3-Ethylbenzothiazoline-6-sulphonic Acid) Radicals, and TPC (Total Phenolic Content)

The most active extract towards the DPPH radical was the methanol extract of *M. edule*, with an IC_50_ value of 0.1 mg/mL, similar to the one obtained with the synthetic antioxidant butylated hydroxytoluene (BHT) used as a standard (IC_50_ = 0.1 mg/mL; Table 1). The species *M. edule* L. (syn. *Carpobrotus edulis*, sourfig or highway iceplant) is an edible facultative halophyte with a long tradition of use in the South African folk medicine against fungal and bacterial infections of the skin and mouth, dermal itching caused by insect bites, for treating wounds and burns and also for the treatment of sinusitis, diarrhea, infantile eczema and tuberculosis [7,8]. Sourfig is native to the coastal areas of South Africa and was introduced in southern and western Europe, including the Algarve, for soil stabilization and landscaping along railroad tracks and in sand banks. Because of its highly successful reproduction and dispersal capacity, sourfig became an invasive species in several parts of the world, including Europe, Australia, California and the Mediterranean. As physical (e.g., manual removal of plants) and chemical (*i.e.*, use of herbicides) control are currently being used to decrease its populations, a more useful approach should include the use of the harvested plant as a source of beneficial secondary metabolites. The results obtained in this work are in accordance with previous *in vitro* studies showing that *M. edule* is endowed with strong antioxidant compounds, possibly of a polyphenolic nature [9,10,11,12]. Those properties suggests that biomass from *M. edule* could be used as source of antioxidants, thus contributing to the sustainable control of this invasive species.

A high radical scavenging activity (RSA) against DPPH^•^ was also obtained with the methanol extract of *J. acutus* (IC_50_ = 0.4 mg/mL), and the diethyl ether extracts of *J. acutus* (IC_50_ = 0.2 mg/mL) and *A. macrostachyum* (IC_50_ = 0.3 mg/mL; Table 1). Concerning the 2,2′-azino-*bis* (3-ethylbenzothiazoline-6-sulphonic acid) (ABTS) free radical, the lowest IC_50_ values were obtained in the diethyl ether extract of *J. acutus* (IC_50_ = 0.4 mg/mL) and *H. portulacoides* (IC_50_ = 0.9 mg/mL, Table 1). The species *J. acutus* is traditionally used for the treatment of infection and inflammation [13], and is endowed with antioxidant compounds, such as phenolics (e.g., 8,8′-bidehydrojuncusol) and flavones (e.g., luteolin), which were isolated from methanol extracts of the rhizomes [14]. The C3 shrub *A. macrostachyum* L. is also considered to be a medicinal plant [15], containing metabolites with RSA of the DPPH radical, and also iron reducing and copper chelating activities [12]. *H. portulacoides* is an obligate halophyte able to maintain growth in salinities up to 1 M NaCl [16]. Leaf waxes of *H. portulacoides* contain long chain chloroalkanes [17], and volatile organic compounds were detected in the root exudates [18]. Moreover, in a recent work it was found that the lipophilic fraction of leaves and stems of *H. portulacoides* was mainly composed of long chain aliphatic acids, alcohols and sterols, while the major component of the roots was a triterpenic ketone, whereas the leaves had a high content of phenolic compounds, mostly sulfated flavonoids [19]. However, to the best of our knowledge, there are no reports of the biological activity of this species.

The extracts with the highest total phenolic content (TPC) were the methanol extract of *M. edule* (147 mg gallic acid equivalents (GAE)/g DW) and the diethyl ether extract of *J. acutus* (93 mg GAE/g DW), which was correlated with a high RSA (Table 1). This is in agreement with several reports of positive correlations between the content of phenolics of halophyte extracts and its capacity to scavenge free radicals [10,20,21]. However, in other extracts and species no correlation between these parameters was observed, similar to the findings of Conforti *et al.* [22] in hydroalcoholic extracts of Mediterranean dietary plants. This suggests that the RSA of these samples might be due to combined action of phenolic compounds with other components such as peptides and organic acids [23].

**Table 1:** Radical scavenging activity (RSA) on 1,1-diphenyl-2-picrylhydrazyl (DPPH) and 2,2′-azino-*bis*(3-ethylbenzothiazoline-6-sulphonic acid) (ABTS) radicals (IC_50_, mg/mL) and total phenolic content (TPC) (mg gallic acid equivalents (GAE)/g DW) of extracts of *A. macrostachyum*, *P. coronopus*, *M. edule*, *J. acutus* and *H. portulacoides.*

Species/Compound	Extract	DPPH	ABTS	TPC
*A. macrostachyum*	Hexane	5.0 ± 0.1 ^c^	9.6 ± 0.5 ^h^	39 ± 0.8 ^h^
	Diethyl ether	**0.3** ± 0.0 ^a^	2.7 ± 0.1 ^d,e^	33 ± 1.6 ^g^
	Chloroform	0.6 ± 0.1 ^a^	2.0 ± 0.0 ^c,d,e^	33 ± 0.4 ^g^
	Methanol	3.4 ± 0.1 ^b,c^	5.2 ± 0.2 ^g^	72 ± 0.5 ^k^
	Water	>10	>10	6.6 ± 0.2 ^a^
*P. coronopus*	Hexane	>10	>10	5.8 ± 0.2 ^a^
	Diethyl ether	8.9 ± 0.5 ^d^	>10	16 ± 0.5 ^b,c^
	Chloroform	>10	>10	13 ± 0.2 ^b^
	Methanol	0.9 ± 0.1 ^a^	1.1 ± 0.1 ^a,b^	103 ± 1.8 ^m^
	Water	4.0 ± 1.1 ^c^	2.1 ± 0.0 ^d,e^	28 ± 0.2 ^f^
*M. edule*	Hexane	5.3 ± 0.6 ^c^	>10	4.5 ± 0.3 ^a^
	Diethyl ether	1.8 ± 0.1 ^a,b^	2.9 ± 0.1 ^e^	22 ± 0.8 ^e^
	Chloroform	>10	5.3 ± 0.0 ^f^	56 ± 0.7 ^j^
	Methanol	**0.1** ± 0.0 ^a^	2.0 ± 0.0 ^c,d,e^	**147** ± 0.6 ^n^
	Water	1.1 ± 0.3 ^a^	7.9 ± 0.2 ^g^	52 ± 1.5 ^j^
*J. acutus*	Hexane	4.3 ± 0.3 ^c^	8.6 ± 0.3 ^g,h^	17 ± 0.3 ^c,d^
	Diethyl Ether	**0.2** ± 0.0 ^a^	**0.4** ± 0.0 ^a^	**93** ± 0.5 ^l^
	Chloroform	>10	1.8 ± 0.3 ^b,c,d^	20 ± 0.3 ^g,h^
	Methanol	0.4 ± 0.0 ^a^	1.8 ± 0.1 ^b,c,d^	35 ± 0.2 ^i^
	Water	>10	2.6 ± 0.1 ^d,e^	35 ± 1.0 ^g^
*H. portulacoides*	Hexane	>10	>10	5.5 ± 0.1 ^a^
	Diethyl ether	>10	**0.9** ± 0.0 ^a,b^	55 ± 0.7 ^j^
	Chloroform	>10	4.0 ± 0.1 ^f^	13 ± 0.3 ^b^
	Methanol	>10	>10	15 ± 0.1 ^b,c^
	Water	>10	>10	21 ± 0.5 ^d,e^
BHT *	-	0.1 ± 0.0	0.1 ± 0.0	-

Values represent the mean ± standard error of mean (SEM) of at least three experiments performed in triplicate (*n* = 9), * Butylated hydroxytoluene (BHT, E320): positive control. For the same column, different letters in the same column are significantly different (Tukey HSD test, *p* < 0.05). Values in bold indicate high activity.

### 2.2. Anti-Inflammatory Activity

To evaluate the *in vitro* anti-inflammatory activity of the extracts, the effect of nontoxic concentrations on the nitric oxide (NO) production was measured in lipopolysaccharide (LPS) stimulated RAW264.7 macrophage cells. LPS is in this context an endotoxin responsible for septic shock syndrome, which stimulates the production of inflammatory mediators such as NO, a radical often associated with the expression of pro-inflammatory proteins, namely iNOS and cyclooxygenase (COX-2) [24]. Thus, a reduction in NO production is indicative of the potential of the extracts to attenuate an inflammatory response.

A significant decrease in NO production was observed upon incubation of macrophages with LPS and the chloroform extract of *H. portulacoides* (IC_50_ = 109 µg/mL) as well as the hexane extract of *P. coronopus* (IC_50_ = 98 µg/mL; Table 2), which suggests the presence of compounds with anti-inflammatory potential in these samples. This is the first report describing the anti-inflammatory potential of *H. portulacoides* and *P. coronopus*. However, other species of the *Plantago* genus, namely *P. altissima* and *P. lanceolata*, have already been described as containing anti-inflammatory molecules capable to inhibit COX-1 and 12-lipoxygenase (12-LOX) [25]. Interestingly, the application of the water extract of *M. edule* (3.9–125 µM) significantly increased the NO production (Table 2), which suggests that this extract contains compounds able to stimulate the response of RAW264.7 macrophages against LPS stimulation. Ordway *et al.* [26] observed that the methanol extract of *M. edule* exhibited an immunomodulatory effect, since it was able to stimulate THP-1 human monocyte-derived macrophages to kill ingested *Staphylococcus aureus*, and to promote the release of lymphokines associated with cellular immune functions, namely interferon gamma (IFN-γ). Although the compounds responsible for those activities are still unknown, such bioactivity could be useful for tuning and modification of the responses from the immune system, through the stimulation of the macrophage-mediated immune response. In this way, the aqueous extract of *M. edule* could be used as an immunostimulant, aiding infection resolution, in accordance with its *in vitro* antibacterial potential as described by Martins *et al.* [27].

### 2.3. Cytotoxic Activity

**Figure 1:**
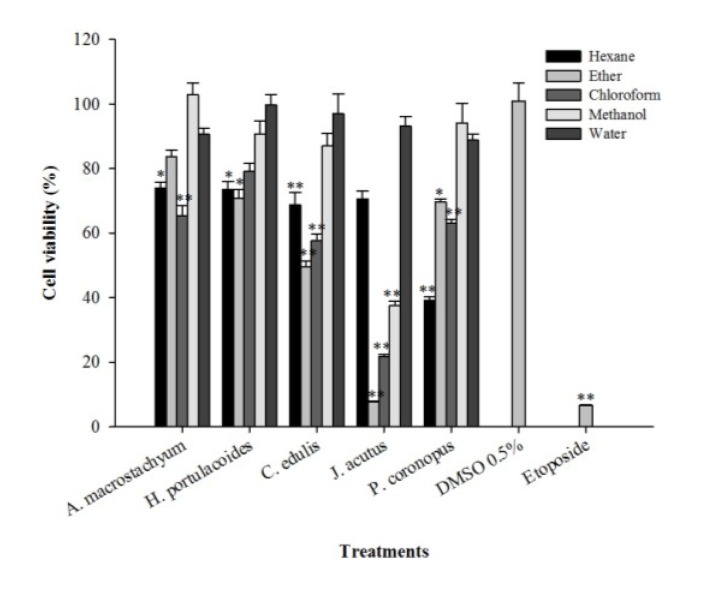
Effect of the application of different extracts of halophytes and etoposide on HepG2 cellular viability. Results are expressed as % of viability relative to a control containing dimethyl sulfoxide (DMSO) (0.5%, *v*/*v*). Solid and errors bars represent the average and SEM, respectively (*n* = 12). Significant differences between control and treated cells are indicated as follows: * *p* < 0.01, ** *p* < 0.001.

The evaluation of the cytotoxic activity of the extracts was made *in vitro* through the application of the samples to a human hepatocarcinoma cell line (HepG2) for 72 h at a concentration of 125 µg/mL, followed by determination of cell viability using the 3-(4,5-dimethylthiazol-2-yl)-2,5-diphenyltetrazolium bromide (MTT) assay. A significant reduction in HepG2 cell viability was observed after application of the hexane extract of *P. coronopus* (39%) and the diethyl ether and chloroform extracts of *M. edule* (50% and 58% of cellular viability, respectively; Figure 1).

**Table 2:** Nitric oxide (NO) production (% and IC_50_ values, µg/mL) relative to lipopolysaccharide (LPS)-stimulated RAW264.7 macrophages incubated with non-toxic concentrations of extracts of *A. macrostachyum*, *P. coronopus*, *H. portulacoides*, *J. acutus* and *M. edule*.

Species/Compound	Extract	3.9 µg/mL	7.8 µg/mL	15.6 µg/mL	31.2 µg/mL	62.5 µg/mL	125 µg/mL	IC_50_
*A. macrostachyum*	Water	118 ± 3 ***	117 ± 6	99 ± 4	103 ± 3	87 ± 4	77 ± 3 **	n.d.
*P. coronopus*	Hexane	-	-	-	-	56 ± 6 ***	47 ± 3 ***	98 ± 4.0
	Chloroform	102 ± 5	97 ± 7	93 ± 8	90 ± 11	-	-	n.d
	Methanol	-	-	-	96 ± 3	97 ± 2	79 ± 3 ***	n.d
	Water	112 ± 1	111 ± 2	110 ± 1	111 ± 2	103 ± 3	98 ± 3	n.d
*H. portulacoides*	Chloroform	-	-	-	83 ± 3	73 ± 3 **	43 ± 1 ***	109 ± 2.5
*J. acutus*	Hexane	102 ± 3	-	-	-	-	-	n.d
	Chloroform	103 ± 7	-	-	-	-	-	n.d
*M. edule*	Methanol	112 ± 4	114 ± 5	103 ± 3	115 ± 6	89 ± 5	111 ± 3	n.d
	Ether	106 ± 3	104 ± 2	96 ± 2	-	-	-	n.d
	Chloroform	103 ± 2	89 ± 4	83 ± 6.6	-	-	-	n.d
	Water	144 ± 2 ***	128 ± 6 **	169 ± 6 ***	137 ± 7 ***	137 ± 6 ***	98 ± 6	n.d
L-NAME *	-	-	-	-	-	-	-	29 ± 2.1

Values represent the mean ± SEM of at least three experiments performed in triplicate (*n* = 9). Statistical significance in NO production between cells containing DMSO (0.5%, *v*/*v*) diluted in culture medium and those treated with halophyte extracts are indicated as follows: * *p* < 0.01, ** *p* < 0.001; *** *p* < 0.0001; -, not tested; n.d, not determined; l-NAME: NG-nitro-l-arginine methyl ester: positive control. The IC_50_ values were calculated as described on the materials and methods section, from a minimum of five concentrations.

The use of several *Plantago* species by humans against cancer has been reported by different authors, and includes the species *P. coronopus*, *P. lanceolata*, *P. major*, *P. ovate* and *P. hirtella* [28]. A methanol extract of *P. coronopus* significantly reduced the viability of human breast (MCF-7) and melanoma (UACC-62) cell lines [29]. In the same way, several compounds with antiproliferative activity against mouse T-cell lymphoma cells were isolated from *M. edule*, namely β-amyrin, uvaol, oleanolic acid, monogalactosyldiacylglycerol, catechin, epicatechin and procyanidin B5 [30].

The best result was achieved with the diethyl ether extract of *J. acutus*, which reduced cell viability to 7.7%; similar to the results obtained with the positive control compound, the drug etoposide (Figure 1). The chloroform and methanol extracts of *J. acutus* also showed low tumor cell viabilities of 22% and 37%, respectively (Figure 1). *Juncus* is the largest genus in the Juncaceae family comprising more than 200 species that usually grow in maritime environments, such as salt marshes, or in badly drained soils under different climatic conditions [31]. It has been claimed that several species belonging to the *Juncus* genus exhibit medicinal properties: the medulla of *J. effusus* (L.) is used as an antipyretic and sedative agent whereas the rhizomes of *J. maritimus* are recommended for insomnia [31]. The species *J. rigidus* has diuretic effects and is useful in the treatment of stomach disorders [2]. Leaves from *J. acutus* are used in the province of Almeria (Spain) to treat warts [32], in oriental traditional medicine the seeds of *Juncus* sp. are used for the treatment of diarrhea and fruits are used in infusions to alleviate cold symptoms [31].

Several biological activities have been ascribed to extracts made from different species of *Juncus*, namely cytotoxicity, antitumoral, anti-eczematic, anti-inflammatory, anti-algal, antioxidant and hepatoprotective [2,31]. *Juncus* species are known to contain secondary metabolites of different classes, namely coumarins, flavonoids, sterols, terpenes, phenolic acids, stilbenes, carotenoids and phenanthrenes [2,31]. The rhizomes of *J. acutus* are known to contain phenanthrenoids with anti-inflammatory activity [33], while the aerial parts are endowed with phenolic glycosides displaying anti-eczematic activity [13]. Regarding *in vitro* antitumoral activity, it was shown that a hydroalcoholic extract of the tops of the species *J. roemerianus* was active against the National Cancer Institute's murine P-388 lymphocytic leukemia [34]. However, to the best of our knowledge, until now there has been no information about the cytotoxic activity of *J. acutus* towards human tumoral cells.

Given the promising results obtained with the diethyl ether extract of *J. acutus*, the IC_50_ values and the selectivity index (SI) towards cells of non-tumoural origin (S17, murine bone marrow) were also determined and are summarized in Table 3. According to dos Santos *et al.* [35], natural extracts are considered promising sources of antitumoral compounds when they exhibit IC_50_ values lower than 30 µg/mL. That was the case of the *J. acutus* extract, with an IC_50_ value of 6.2 µg/mL in HepG2 cells, significantly lower than that obtained in the S17 cell line (IC_50_, 34.4 µg/mL, *p* < 0.05). Moreover, the SI displayed by *J. acutus* (5.5) was similar to the one observed for etoposide (SI = 5.4, Table 3).

**Figure 2:**
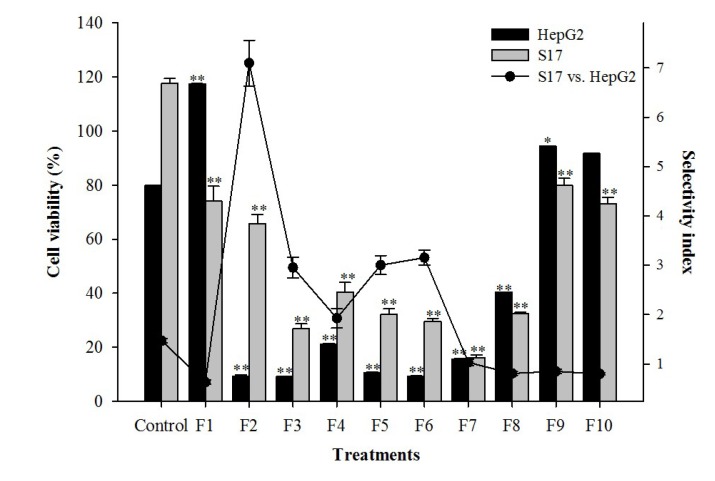
Effect of the application of the fractions obtained from *J. acutus*, at a concentration of 15 µg/mL on HepG2 and S17 cell lines, expressed as cell viability (*bar chart*) and selectivity (*scatter line*). Results are expressed as mean ± SEM of at least three experiments performed in triplicate (*n* = 9). Significant differences in viability between control and treated cells are indicated as follows: * *p* < 0.01, ** *p* < 0.001.

In order to identify the compound responsible for the cytotoxic activity, the extract from *J. acutus* was submitted to a bio-guided fractionation, affording 10 fractions from which fractions 2 to 8 were cytotoxic to HepG2 and S17 cells (Figure 2). Fraction **2** displayed the highest selectivity against HePG2 cells (SI = 7.1), and was further fractionated until an active and apparently pure compound was obtained. The chemical structure of the isolated compound was established using spectral data obtained with FT-IR, ^1^H-NMR, ^13^C-NMR and mass spectrometry.

**Table 3:** IC_50_ values (µg/mL) and selectivity index (SI) of the diethyl ether extract of *J. acutus* and of etoposide, on a human tumoral cell line (HepG2) and on murine non-tumoral cells (S17).

Treatment	IC_50_ values		SI
	HepG2	S17	HepG2
Extract	6.2 ± 0.3 *	34 ± 2.2	5.5
Etoposide	1.9 ± 0.1	10 ± 0.01	5.4

Values represent the mean ± SEM of at least three experiments performed in triplicate (*n* = 9); * Indicates significant differences (*p* < 0.01) as compared with S17 cells.

**Figure 3:**
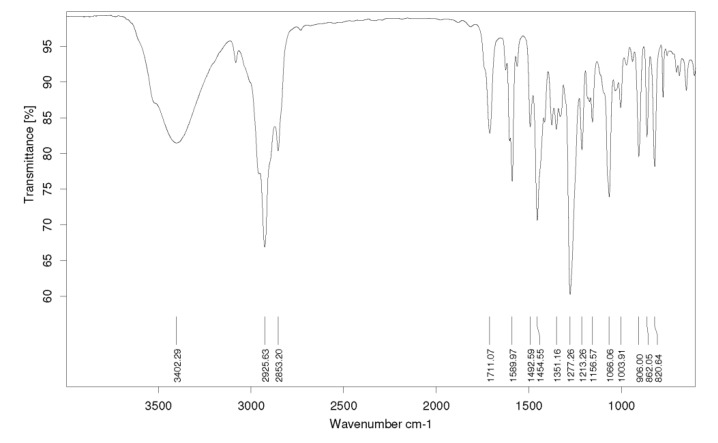
FTIR spectra of the pure isolated compound.

The FT-IR spectrum showed a characteristic broad absorption peak at 3402 cm^−1^, assigned to a hydroxyl group and at 1711 cm^−1^ characteristic of aromatic stretching and a small sharp stretching at about 3100 cm^−1^ (*sp*^2^ hybridized C-H) (Figure 3). HR-ESIMS analysis ([M + H]^+^) indicated a compound with the molecular formula C_18_H_19_O (calc. M.W. 251.1430, det. M.W. 251.1427).

**Figure 4:**
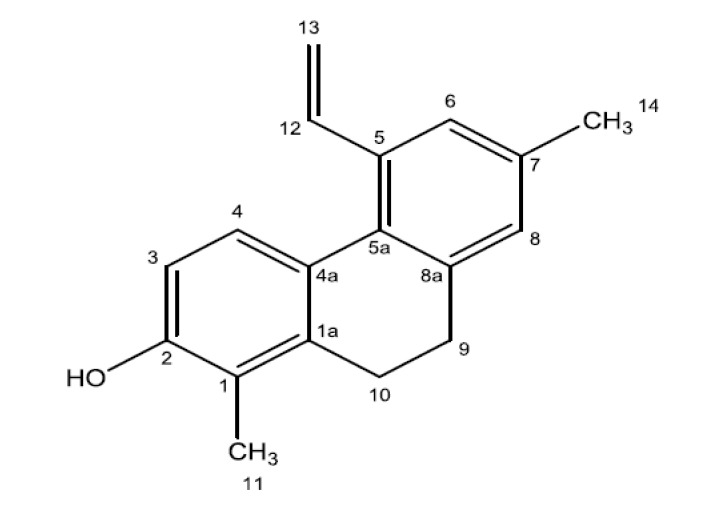
Chemical structure of juncunol (1,7-dimethyl-5-vinyl-9,10-dihydrophenanthren-2-ol).

The chemical shifts from ^1^H and ^13^C-NMR spectra were consistent with the compound juncunol (Figure 4) [36].

This compound was previously identified in *J. acutus* and *J. roemerianus* [36,37,38,39], and displayed phytotoxicity against the microalga *Selenastrum capricornutum* [38], but low activity against the growth of *Agrobacterium tumefaciens* using the potato disc assay, and also in the brine shrimp assay [36,37]. However, to the best of our knowledge, nothing was known until now about the *in vitro* cytotoxic activity of juncunol against human cancer cells. The cytotoxicity of pure juncunol was further tested in different tumoral cell lines and the selectivity towards S17 and mTEC (mouse thymic epithelial) cells was assessed (Table 4).

**Table 4:** Effect of the application of juncunol and etoposide on the viability of tumoral (HepG2) and non-tumoral cell lines (S17 and mTEC), expressed as IC_50_ values (µM/mL), and selectivity index (SI).

Cell lines	Etoposide			Juncunol		
	IC_50_	SI: S17	SI: mTEC	IC_50_	SI: S17	SI: mTEC
*Tumor cell lines*						
HepG2	2.4 ± 0.2 ^a^	7.5 ± 0.4 ^B^	33 ± 2.3 ^G^	18 ± 1.2 ^b,c^	20 ± 1.4 ^F^	18 ± 1.2 ^E,F^
MDA-MB-468	11 ± 0.7 ^c^	1.6 ± 0.1 ^A^	6.1 ± 0.7 ^B^	22 ± 2.0 ^b,c^	17 ± 1.7 ^E,F^	15 ± 1.6 ^D,E^
HeLa	7.1 ± 0.2 ^b^	2.5 ± 0.1 ^A^	10 ± 0.8 ^C^	27 ± 2.8 ^c^	13 ± 1.2 ^C,D^	12 ± 1.3 ^C,D^
*Non-tumor cell lines*						
S17	17 ± 0.2 ^d^	-	-	367 ± 3.2 ^g^	-	-
mTEC	75 ± 3.7 ^e^	-	-	344 ± 6.0 ^f^	-	-

Values represent the mean ± SEM of at least three experiments performed in triplicate (*n = 9*). In the same column, values followed by different letters (a–g for IC_50_ values and A–G for SI) are significantly different (Tukey HSD test, *p* < 0.05). HepG2: human hepatocarcinoma; MDA-MB-468: human breast carcinoma; HeLa: human cervical carcinoma; S17: murine stromal; mTEC: mouse thymic epithelial.

In addition to HepG2 cells, juncunol was able to significantly reduce the viability of human breast and cervical cell lines, while maintaining a high SI towards S17 (SI values of 17 and 14 respectively) and mTEC cell lines (SI values of 16 and 13, respectively; Table 4). Juncusol, a dihydrophenanthrene closely related to juncunol was isolated from *J. roemerianus* and was demonstrated to significantly reduce the viability of different cell lines, such as human epidermoid carcinoma of the nasopharynx (NCI 90 KB: IC_50_ = 0.3 µg/mL), B-16 mouse melanoma (IC50 = 12.5 µg/mL) and also of L-1210 mouse lymphocytic leukaemia (IC_50_ = 12.5 µg/mL) [34], but no assays were performed in order to establish the selectivity of that compound. The IC_50_ values obtained in our work (HePG2 = 4.6 µg/mL; MDA-MB-468 = 5.6 µg/mL and HeLa = 6.9 µg/mL) were lower than those obtained with juncusol suggesting a higher *in vitro* cytotoxicity of juncunol. Nevertheless, one must bear in mind that different cell lines and methodologies were tested.

Taken together, our results indicate that juncunol is a good candidate for further studies on its potential use as an anticancer drug or as a valuable lead compound for the semi-synthesis or total synthesis of effective novel drugs. Assays are currently being conducted in order to elucidate its mode of action.

## 3. Experimental Section

### 3.1. Reagents

DPPH, sodium nitrite, LPS from *Escherichia coli,* sulphanilamide, *N*-(1-naphthyl)-ethylenediamine dihydrochloride (NED) and MTT were purchased from Sigma-Aldrich (Steinheim, Germany). Merck (Darmstadt, Germany) supplied Folin-Ciocalteau (F-C) phenol reagent and phosphoric acid. Lonza (Leuven, Belgium) provided Dulbecco’s Modified Eagle’s Medium (DMEM), fetal bovine serum (FBS), trypsin, l-glutamine and penicillin/streptomycin. Additional reagents and solvents were obtained from VWR International (Leuven, Belgium).

### 3.2. Plant Material and Preparation of the Extracts

Five species of halophytes were collected in the south of Portugal, near Faro beach and Ludo, in May 2010, namely *A. macrostachyum*, *M. edule*, *J. acutus*, *P. coronopus* and *Halimiones portulacoides*. Taxonomical classification was confirmed by Dr. Manuel J. Pinto (National Museum of Natural History, University of Lisbon, Botanical Garden, Portugal). Voucher specimens are being kept in a herbarium in the MarBiotech laboratory. Samples were oven dried for three days at 40 °C and powdered, and the extracts were prepared sequentially as follows: the dried powder was mixed with hexane (1:10, *w*/*v*) and homogenized during 2 min. using a disperser IKA Ultra-Turrax T10B, at room temperature (RT). Samples were then centrifuged (10 min, 5000× *g*, RT), and the supernatants were recovered. The extraction was repeated three more times and the supernatants were combined and filtered (Whatman No, 4). The remaining biomass was consecutively extracted with diethyl ether, chloroform, methanol and water. The organic extracts were evaporated under reduced pressure, and aqueous extracts were freeze dried. Dried extracts were weighed, dissolved in DMSO to obtain a final concentration of 50 mg/mL and stored at 4 °C.

### 3.3. RSA by the DPPH^•^ Assay

The antioxidant activity was assessed by the DPPH^•^ assay, according to the method of Brand-Williams *et al.* [40], as described by Moreno *et al.* [41]. Samples (22 µL) at concentrations ranging from 0.125 to 10 mg/mL were mixed with 200 µL of a methanol DPPH^•^ solution (120 µM) in 96-well microplates and incubated for 30 min at RT, in the dark. Absorbance was measured at 517 nm using a Biotek Synergy 4 microplate reader and results were expressed as antioxidant activity (%), relative to a control containing DMSO and as half maximal inhibitory concentration (IC_50_, mg/mL). BHT (E320) was used as the positive control at the same concentrations of the biological samples.

### 3.4. RSA by the ABTS^•+^ Assay

The RSA against ABTS**^•+^** was evaluated according to Re *et al.* [42]. A stock solution of ABTS**^•+^** (7.4 mM) was prepared in potassium persulfate (2.6 mM), and left in the dark for 12–16 h at RT. The ABTS**^•+^** solution was then diluted with ethanol to get an absorbance of 0.7 at 734 nm (Biotek Synergy 4). The samples (10 µL), at concentrations ranging from 125 to 1000 µg/mL, were mixed with 190 µL of ABTS**^•+^** solution in 96-well microplates, and after 6 min of incubation the absorbance was measured at 734 nm (Biotek Synergy 4, Biotek, Winooski, VT, USA). Results were presented as antioxidant activity (%) relative to a control containing DMSO, and as IC_50_ values (mg/mL). BHT was used as a positive control at the same concentrations of the extracts.

### 3.5. Total Phenolic Content (TPC)

The TPC of the extracts was determined by the F-C colorimetric assay [43]. The experiments were performed in 96-well plates: 5 µL of each extract at a concentration of 10 mg/mL were mixed with 100 µL of diluted F-C (1:10, *v*/*v* in water). After 5 min, 100 µL of a sodium carbonate solution (75 g/L, in water) were added, and the mixture was incubated at RT for 90 min in the dark. Absorbance was measured at 725 nm in a microplate reader (Biotek Synergy 4). TPC was calculated based on a standard curve of gallic acid and the results were expressed as milligrams of gallic acid equivalents per gram of dry weight (mg GAE/g DW).

### 3.6. Cell Culture

The HepG2 cell line (human hepatocellular carcinoma) was kindly provided by Dr. Vera Marques, while S17 cells (murine bone marrow stromal), mTEC (mouse thymic epithelial) and MDA-MB-468 (human breast carcinoma) cells were provided by Dr. Nuno Santos (CBME, University of Algarve, Faro, Portugal). The murine leukemic monocyte-macrophage cell line (RAW264.7) was obtained from Faculty of Pharmacy and Center for Neurosciences and Cell Biology (University of Coimbra, Coimbra, Portugal). All cell lines were maintained in DMEM culture medium supplemented with 10% heat-inactivated FBS, 1% l-glutamine (2 mM), and 1% penicillin (50 U/mL)/streptomicin (50 μg/mL), and were maintained at 37 °C in a humidified atmosphere with 5% CO_2_.

### 3.7. Quantification of NO

Exponentially growing RAW264.7 cells were plated at 2.5 × 10^5^ cells/well in 96-well tissue plates and allowed to adhere overnight. Afterwards, cells were treated with concentrations of the extracts allowing cellular viability higher than 80%, in serum- and phenol-free culture medium, for 24 h together with LPS (100 ng/mL) [44]. Control cells were treated with DMSO at the highest concentration used in test wells (0.5%), and NO production in cell culture medium was measured spectrophotometrically by the Griess method [45]. In brief, 100 µL of the culture supernatants were mixed with 100 µL of Griess reagent (1% (*w*/*v*) sulphanilamide + 0.1% of NED and 2.5% (*v*/*v*) phosphoric acid), incubated for 20 min at RT in the dark, and absorbance was measured at 540 nm on a microplate reader (Biotek Synergy 4). The NO concentration was determined using a calibration curve prepared with several known concentrations (1.7, 3.1, 6.2, 12.5, 25, 50 and 100 µM) of sodium nitrite as standard. Results were expressed as NO production (%) relative to LPS-stimulated RAW264.7 cells, and as IC_50_ values (µg/mL).

### 3.8. Cell Viability Assay

Exponentially growing HepG2 and S17cells were plated in 96-well tissue plates at a density of 5 × 10^3^ cells/well and incubated for 24 h. Then, extracts were applied at various concentrations (3.9, 7.8, 15.6, 31.2, 62.5 and 125 µg/mL) for 72 h. Control cells were treated with DMSO at the highest concentration used in test wells (0.5%), and cell viability was determined by the MTT colorimetric assay [46]. Briefly, 2 hours prior to the end of the incubation period 20 μL of MTT (5 mg/mL in PBS) were added to each well and further incubated at 37 °C. Then, 150 μL of DMSO was added to each well in order to dissolve the formazan crystals and absorbance was measured at 590 nm (Biotek Synergy 4). Results were expressed in terms of cell viability (%) and IC_50_ values (µg/mL). The selectivity index (SI) of the extracts was estimated using the following equation: SI = VNT/VT, where VNT and VT indicate cell viability on non-tumoral cells (S17) and tumoral cells (HepG2), respectively [47].

### 3.9. Bioguided Fractionation and Isolation of the Bioactive Compound

The active crude extract of *J. acutus* (5 g) was subjected to silica gel (120 mesh) column chromatography (25 cm × 2 cm i.d), and eluted with different proportions of *n*-hexane, a mixture of *n*-hexane and ethyl acetate (90:10; 85:15; 4:1; 75:25; 7:3; 3:2 and 1:1), ethyl acetate and a mixture of chloroform and methanol (1:1). Fractions with similar TLC profiles using GF254 (Merck) as stationary phase and *n*-hexane/EtOAc 3:1 and 4:1 as mobile phase, were pooled yielding 10 fractions, which were tested for cytotoxic activity and selectivity as described on the Section 3.8. Based on the results, fraction **2** (0.76 g) was selected and subjected to a new silica 60 mesh column chromatography (14 cm × 2 cm i.d) and sequentially eluted with *n*-hexane and a mixture of *n*-hexane and ethyl acetate (98:2 and 95:5) affording 3 fractions, which were again evaluated for cytotoxicity. Fraction **3** (100 mg), an orange solid powder, was active and contained a pure compound, which was stored at 4 °C until further use.

### 3.10. Spectral and Chromatographic Analysis

IR spectra were recorded on a Bruker spectrophotometer (Bruker, Coventry, UK) in a range of 500–4000 cm^−1^. GC-MS analysis was performed using Agilent 6890N Gas Chromatograph connected to Agilent 5973 Mass Spectrometer (Agilent, Santa Clara, CA, USA) operated at 70 eV. The isolated compound was dissolved in CDCl_3_ (99.8% D) and analyzed at 27 °C at 600.03 MHz for ^1^H and 150.88 MHz for ^13^C. NMR-spectra were acquired using a 600 MHz Bruker Avance III HD equipped with a cryogenically cooled 5 mm dual probe optimized for ^13^C and ^1^H. Proton spectra were acquired using 30°-pulses, a spectral width of 12 kHz, collecting 16 scans with a length of 65,536 data points with a relaxation delay of 1.0 s. Carbon spectra were acquired with 30°-pulses, a spectral width of 36 kHz, collecting 256 scans with a length of 65,536 data points and with a relaxation delay of 2.0 s. FIDs were exponentially multiplied with a line broadening factor of 0.3 Hz (^1^H) and 1.0 Hz (^13^C) before Fourier transform. Signals were assigned using information obtained by COSY, HSQC, HMBC (65 ms and 100 ms mixing time) experiments. Standard parameters and sequences were used as delivered with the software Topspin (version 3.2, Bruker). HR-ESIMS were performed on a microQTOF-QII mass spectrometer (Bruker Daltonik GmbH) equipped with an electrospray ionization interface (ESI). The sample were introduced using a RP-HPLC system (Agilent 1200, Agilent, Santa Clara, CA, USA) employing a steep gradient of acetonitrile in water with 0.1% formic acid. A small portion of the eluate was directed towards the ESI where it was ionized using a capillary voltage of 4100 V, a drying temperature of 200 °C, nebulizer pressure of 2.0 bar and a drying gas flow of 7 L/min. Mass spectra were externally calibrated using a standard of sodium formate clusters introduced just before the analysis.

### 3.11. Statistical Analysis

The results were expressed as mean ± SEM, and the experiments were conducted in triplicate. Analysis of variance (ANOVA) was used to assess differences using the SPSS statistical package for Windows (release 15.0, SPSS INC), and significance between means was analyzed by the Tukey HSD test (*p* < 0.05). The IC_50_ values were calculated by sigmoidal fitting of the data in the GraphPad Prism V 5.0 program (GraphPad Software, La Jolla, CA, USA).

## 4. Conclusions

Halophytes are endowed with a vast array of compounds displaying important biological activities, such as antioxidant, antimicrobial and antitumoral, and therefore could be useful in the prevention and treatment of a variety of diseases, namely cancer, chronic inflammation, atherosclerosis and cardiovascular disorders. In this context, halophytic species have been increasingly considered as an important source of novel active agents with applications in the food and pharmaceutical industries [2]. In this work five maritime halophytic species abundant on the southern coast of Portugal were evaluated for their potential as sources of antioxidant, anti-inflammatory and cytotoxic compounds. *M. edule* and *J. acutus* revealed a strong antioxidant potential and a high content of phenolic compounds, while *H. portulacoides* and *P. coronopus* displayed a high capacity to attenuate NO release in LPS-stimulated macrophages, suggesting a possible anti-inflammatory activity. *J. acutus* exhibited a strong *in vitro* selective cytotoxic activity against HepG2 cells, a human hepatocarcinoma cell line highly resistant to drugs and toxins [48], and therefore, it was fractionated until the bioactive compound was isolated and identified as juncunol (1,7-dimethyl-5-vinyl-9,10-dihydrophenanthren-2-ol). For the first time, juncunol was shown to display a high selective *in vitro* cytotoxicity towards various human cancer cell lines and hence, can be considered a potential chemotherapeutic agent or a scaffold for the semi-synthesis or total synthesis of effective new anticancer drugs. Assays are currently being conducted in order to elucidate the mechanisms responsible for the cytotoxic activity displayed by juncunol.
